# Decreased mTOR signalling reduces mitochondrial ROS in brain via accumulation of the telomerase protein TERT within mitochondria

**DOI:** 10.18632/aging.101089

**Published:** 2016-10-22

**Authors:** Satomi Miwa, Rafal Czapiewski, Tengfei Wan, Amy Bell, Kirsten N. Hill, Thomas von Zglinicki, Gabriele Saretzki

**Affiliations:** ^1^ Institute for Cell and Molecular Biosciences, Newcastle Institute for Ageing, Campus for Ageing and Vitality, Newcastle University, Newcastle upon Tyne, NE4 5PL, UK

**Keywords:** telomerase, brain, aging, rapamycin, dietary restriction

## Abstract

Telomerase in its canonical function maintains telomeres in dividing cells. In addition, the telomerase protein TERT has non-telomeric functions such as shuttling to mitochondria resulting in a decreased oxidative stress, DNA damage and apoptosis. TERT protein persists in adult neurons and can co-localise to mitochondria under various stress conditions. We show here that TERT expression decreased in mouse brain during aging while release of reactive oxygen species (ROS) from the mitochondrial electron transport chain increased. Dietary restriction (DR) caused accumulation of TERT protein in mouse brain mitochondria correlating to decreased ROS release and improved learning and spatial short-term memory. Decreased mTOR signalling is a mediator of DR. Accordingly, feeding mice with rapamycin increased brain mitochondrial TERT and reduced ROS release. Importantly, the beneficial effects of rapamycin on mitochondrial function were absent in brains and fibroblasts from first generation TERT −/− mice, and when TERT shuttling was inhibited by the Src kinase inhibitor bosutinib. Taken together, our data suggests that the mTOR signalling pathway impinges on the mitochondrial localisation of TERT protein, which might in turn contribute to the protection of the brain by DR or rapamycin against age-associated mitochondrial ROS increase and cognitive decline.

## INTRODUCTION

The reverse transcriptase telomerase, best known for its telomere maintaining function in the nucleus, exerts various non-canonical functions in different cellular locations. One of them is the shuttling of the protein part TERT to mitochondria where it has been shown to protect cells from oxidative stress, and reduce mito-chondrial DNA damage and apoptosis [1–3]. Telomerase activity is high in the embryonic brain but downregulated early during human brain development and postnatally in mice [4–7]. However, the telomerase protein TERT has been shown to persist in adult mammalian brains [6, 7–11]. We recently demonstrated that in human hippocampal neurons from late stage Alzheimer's disease (Braak stage 6) a higher amount of TERT protein accumulates within mitochondria compared to those from age-matched healthy brains (Braak stage 0) [10]. We also found that cultivated primary neurons lacking TERT were more sensitive to oxidative stress and to the expression of mutated hyper-phosphorylated tau protein and displayed higher oxidative stress and more cellular damage compared to neurons from wild type, telomerase positive mice [10].

Oxidative stress and mitochondrial dysfunction are well known to increase during aging [12–14] including brain aging [15, 16] and are considered a major cause for cellular senescence [17–19]. During aging, neurons are exposed to high oxidative stress and accumulate dysfunctional mitochondria, damage to DNA, proteins and lipids and changes in energy homeostasis [16, 20]. Recently it became evident that DNA damage during ageing induces a senescent phenotype even in post mitotic cells such as neurons [21]. These changes are associated with significant decrease in neuronal functions such as learning and memory even without obvious pathology in older people. They are further exacerbated in neurodegenerative diseases such as Alzheimer's (AD), Parkinson's (PD), after stroke and brain injury [22–25].

Dietary restriction (DR) is a non-genetic intervention that reproducibly increases mean and maximum lifespan and delays the onset of age-related pathologies in a wide range of tissues including in the brain. The influence of DR on brain aging, neuroplasticity, neuroprotection and cognitive performance has been evaluated in rodent models [26–28]. DR has been shown to improve cognition and spatial memory [29], motor and learning tasks in rodents [30] and slow down the progression of neurodegenerative diseases, such as Alzheimer's disease (AD) [31, 32]. Beneficial effects of DR on mitochondrial function in different tissues have been well documented [see 33 for review] and we have recently shown that DR decreased mitochondrial reactive oxygen species (ROS) production and prevented age-dependent deterioration in mitochondrial coupling by improving the assembly of the electron transport chain complex I in mouse liver and brain [14].

The underlying molecular mechanisms responsible for the beneficial effects of DR are thought to be multifactorial: changes in metabolic signalling via the insulin/IGF axis, sirtuin, AMPK and mTOR pathways have been suggested as mechanisms associated with improved health and longevity after DR [34, 35] and these pathways are also implicated in the aging process [36, 37]. mTOR is a highly conserved serine/threonine protein kinase complex which responds to the levels of nutrients and cellular energy conditions responsible for cell growth, size, metabolism and survival. This pathway is activated by the amount of nutrients in a cell and down-regulated during DR and rapamycin treat-ment. Rapamycin feeding in mice also extended lifespan [38, 39] and reduced mitochondrial ROS production [14, 18]. However, the effects of rapamycin treatment are not identical to that of DR [39, 40].

The aim of this study was to explore whether mitochondrial TERT protein is protective for the brain *in vivo* and whether it plays a role in the beneficial effects of DR and after rapamycin treatment on mitochondrial and brain functions.

Our results demonstrate that DR and the decrease of mTOR activity by rapamycin treatment might be novel and physiologically relevant stimuli to promote mitochondrial TERT localisation specifically in brain *in vivo* resulting in improved mitochondrial function. Importantly, the reduction in mitochondrial ROS release by rapamycin treatment was absent in first generation TERT −/− mice, suggesting that TERT was required for the effect. These results are supported by mechanistic data *in vitro* showing that the decrease of ROS after rapamycin treatment depends on the presence of TERT as well as Src kinase dependent exclusion of TERT from the nucleus. Our data suggest that an increase in mitochondrially localised TERT protein might contribute causally to the beneficial effects of DR and rapamycin in brain.

## RESULTS

### Mitochondrial ROS release increases during aging in brain and is rescued by DR

**Figure 1:**
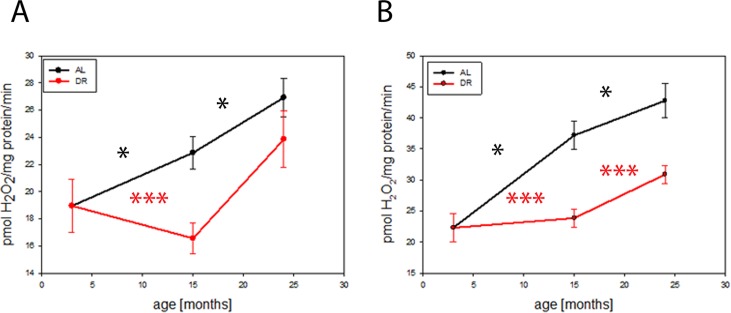
DR delays age-related increase in hydrogen peroxide release from mouse brain mitochondria (**A**) Hydrogen peroxide release from isolated mouse brain mitochondria measured using the Amplex red method at 3, 15 and 24 months under AL and DR conditions: Complex I linked substrate, pyruvate/malate (PM, 5mM) in the presence of rotenone (5μM) (maximum capacity). (**B**) Complex II linked substrate, succinate (4mM). Between 4 (3 months) and 9 (15 months AL) mice per group were used. Data are mean ± S.E.M. Significance for age-related increase was analysed using One way ANOVA compared to 3 months old brains. Differences between AL and DR at each age were compared by t-test. *P<0.05, ***P<0.001.

We performed a long term DR experiment on C57BL6 mice and studied mitochondrial function in brains during aging and the influence of DR. Release of hydrogen peroxide (H_2_O_2_) from complex I of the electron transport chain in isolated brain mitochondria increased with age, determined as its maximum capacity in the presence of the complex I-linked substrate pyruvate plus malate and the complex I inhibitor rotenone (Fig. 1A). There was also an age-dependent increase in the rate of H_2_O_2_ release from mitochondria when they were supplemented with the complex II-linked substrate, succinate (Fig. 1B). DR completely rescued the increase in both parameters until at least 15 months of age and still showed a partial rescue at 24 months, indicating that DR postponed the age-dependent increase in H_2_O_2_ release from brain mitochondria.

### DR improves learning and spatial memory in old mice

In order to correlate the changes in mitochondrial function during aging and in DR with brain function we performed a Barnes maze test for spatial learning and memory on young (10 months) and old AL mice (30 months) as well as old mice on long term DR (33 months). This test included a learning period of 4 days with a test of short term spatial memory on day 5 and long term memory on day 12.

**Figure 2:**
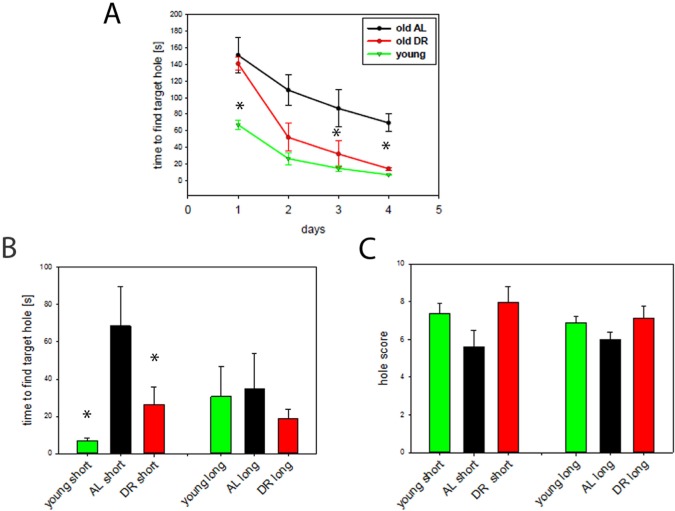
DR improves learning and spatial memory (**A**) Learning behaviour measured as time to find the target hole in a Barnes maze during the training period (day 1 to 4). DR (red, n=12, 33 months), AL (black, n=5, 30 months) and young mice (green, n=7, 10 months,). Statistical significance between groups was tested by One way ANOVA for each day separately. (*P<0.05) (**B**) Short term (short) and long term (long) memory expressed as the time to find the target hole. Young mice n=7, AL, n=5, DR, n=12, One way ANOVA, *p<0.05. (**C**) Short- and long term memory expressed as hole scores for head poking from the same mice as in **A** and **B**.

Fig. 2A demonstrates that old AL and DR mice were significantly slower than young mice in finding the target hole at the start of the experiment (day 1) presumably due to increased anxiety in the old mice. However, from the 3^rd^ day of training it became clear that DR mice learned much better than AL mice, becoming indistinguishable from the young group and significantly different from the AL mice at the end of the training period. In addition, short term memory, measured as the time to locate the target hole on day 5, was superior in DR mice compared to AL mice and reached a similar level as that in young mice (P<0.05) (Fig. 2B, left bars). In contrast, there were no differences in long term memory measured as the time to find the target hole although the groups showed similar tendencies (Fig. 2B, right bars). Equally, hole scores, another measure for spatial memory, showed corresponding tendencies for a decrease with age and an improvement during DR for both short- and long term memory, but did not reach statistical significance (Fig. 2C).

### TERT protein levels in whole mouse brain decrease with age but DR enhances brain mitochondrial TERT abundance

**Figure 3:**
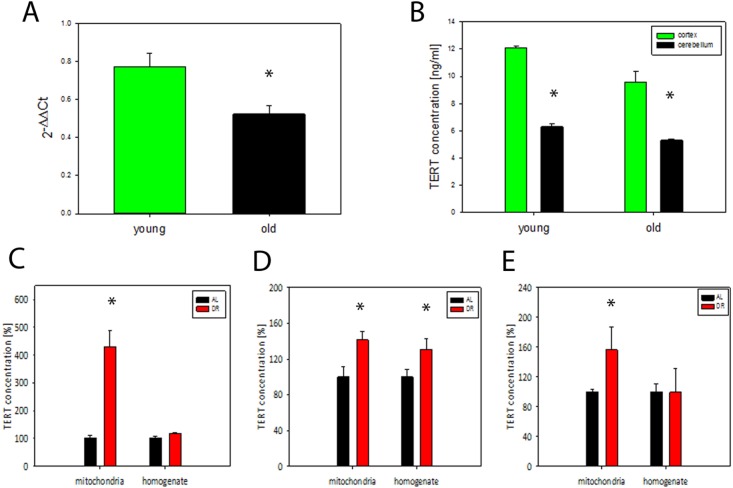
TERT mRNA and protein decrease during brain aging in mice while DR results in TERT accumulation in mitochondria (**A**) TERT mRNA abundance in whole brain tissue from young (4 months) and old (21 months of age) mice (n=3-4 per group), t- test, *P<0.05. (**B**) TERT protein abundance in mouse brain tissue measured separately in cortex and cerebellum in young (6 months) and old (17 months) mice, (n=3 per group), t-test, *P<0.05, (**C-E**) TERT protein abundance in whole brain homogenates and in brain mitochondria in 3 independent DR experiments (as percentage of *ad libitum* fed controls, for details, see Table 1). Exp. 1: n=3 mice per group, exp. 2: n=4 mice per group, exp. 3: n=7 mice per group. t-test, *P<0.05.

We analysed TERT expression in brains from 4 and 21 months old mice using qPCR and found a significant decrease of TERT mRNA at old age (P<0.05, t- test), (Fig. 3A). TERT protein abundance also decreased with age in homogenates from 2 different brain regions (cortex and cerebellum) (Fig. 3B). In agreement with earlier data [6] there was no telomerase activity in brain homogenates from old mice (data not shown). In order to examine whether DR affects TERT abundance and intracellular localisation, we measured TERT abundance in brain homogenates and in matching mitochondrial fractions in 3 independent short term DR experiments (see Table 1 for details) (Fig. 3C-E). In all 3 experiments which varied by age of onset and duration of DR we found a significant increase of TERT protein abundance in mitochondria from brain tissue after DR while there was only in one experiment (number 2, see Table 1) an increase in the whole brain homogenate (Fig. 3C-E). This increase of mitochondrial TERT under DR was tissue-specific: there was no consistently significant increase in TERT abundance in either homogenates (with the exception of exp 1) or isolated mitochondria from livers of the same mice (Suppl. Fig. S1A-C). Moreover, telomerase activity in liver did not increase under DR with the exception of exp 3 (Suppl. Fig. S1D-F).

**Table 1:** Overview of dietary restriction experiments

Experiment	Genotype	Sex	Food restriction[%]	Age at DR onset (months)	Age at end of DR (months)	Duration (months)
1	ICFRa	males	26	15	17.3	2.3
2	C67BL/6	males	40	3	9	6
3	C67BL/6	males	40	3	6	3
4	C67BL/6	males and females	40	3	24	21
5	TERT−/−	males	40	6	22	16

We confirmed the purity of the mitochondrial fractions from brain using Western blot using antibodies against HDAC II (histone deacetylase II, nuclear fraction) and Cox II (cytochrome c oxidase II, mitochondrial fraction, and beta-tubulin for the cytoplasmic fraction (Suppl. Fig. S2). Mitochondrial fractions showed only Cox II signals, while nuclear and cytoplasmic proteins were only detected in the homogenates.

Together, we demonstrate that TERT protein accumulates specifically in brain mitochondria after DR, but not in liver tissue.

### Rapamycin increases mitochondrial TERT abundance in brain and decreases mitochondrial ROS release from wild-type but not TERT−/− mice

**Figure 4:**
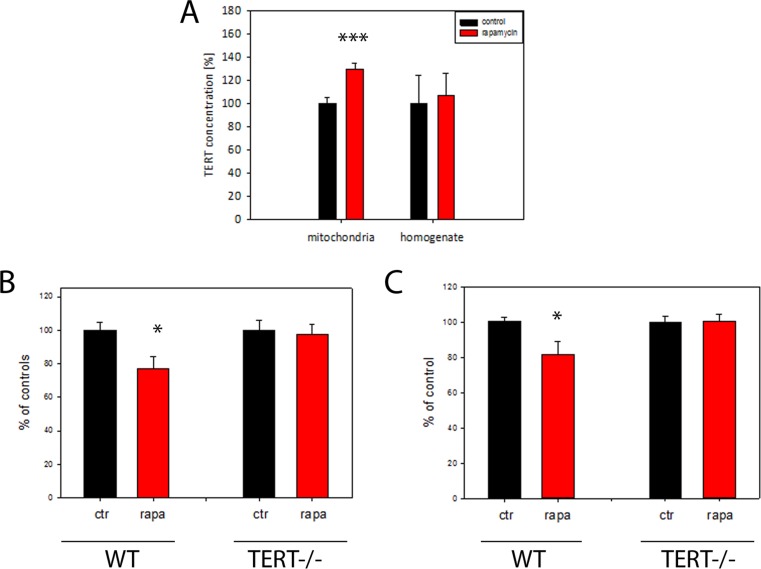
TERT protein is required for the reduction of H_2_O_2_ release from mouse brain mitochondria by rapamycin (**A**) Mouse TERT protein abundance in brain homogenates and isolated mitochondria after 4 months rapamycin treatment. (n=5 per group, t-test, ***p<0.001). (**B**) H_2_O_2_ release (as percentage of controls) from brain mitochondrial complex I (maximum capacity, with complex I-linked substrate, pyruvate plus malate in the presence of rotenone) from wild-type (WT) or first generation TERT−/− mice fed either control or rapamycin-containing diet for 4 months. (n=4-5 per group, t-test: *P<0.05) (**C**) H_2_O_2_ release from brain mitochondria as measured in (**B**), but with the complex II-linked substrate, succinate (4mM). n=4-5 mice per group, t-test *P<0.05.

It has been thought that many, but not all, effects of DR are mediated by suppression of mTOR signalling [35]. In order to explore whether mTOR inhibition is responsible for DR-induced mitochondrial TERT accumulation and ROS reduction, mice were treated for 4 months with the mTOR inhibitor rapamycin. We confirmed suppression of mTOR signalling in brain under short-term DR by showing reduced phosphorylation of mTOR and its downstream target ribosomal protein S6 (experiment 2 in Table 1, Suppl. Fig. S3A, B). 4 months rapamycin treatment did not change the body weight of mice (Suppl. Fig. S3C). As expected, there was significantly less mTORC1 phosphorylation in brains after rapamycin feeding than in controls (Suppl. Fig. S3D). In addition, rapamycin treatment increased TERT transcription in the brain (Suppl. Fig. S3E). Importantly, TERT protein abundance under rapamycin was increased specifically in brain mitochondria but not in tissue homogenate (Fig. 4A).

To test if a higher abundance of mitochondrial TERT causally contributed to a decrease in mitochondrial ROS release via reduced mTOR signalling, we used TERT−/− mice and compared the effects of rapamycin feeding on the rates of mitochondrial H2O2 release with that of wild type mice. Mitochondrial H2O2 release was decreased in brains from rapamycin-treated wild type mice with both complex I and II linked substrates (Fig. 4B, C) but critically, not in TERT −/− mice (Fig. 4B, C). This data suggests that the decrease in mitochondrial ROS release after rapamycin treatment depends on the presence of TERT.

### Rapamycin treatment induces Src dependent shuttling of TERT out of the nucleus causing ROS decrease

**Figure 5:**
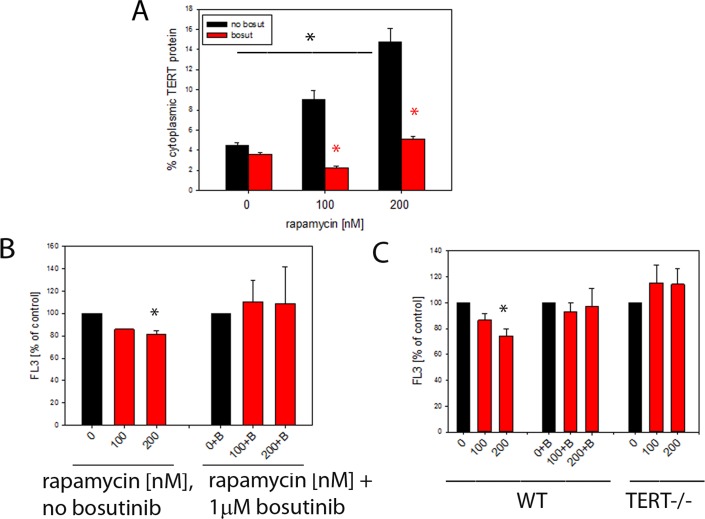
Rapamycin causes nuclear exclusion of TERT and reduces cellular ROS levels in a TERT-dependent fashion (**A**) Extranuclear TERT levels (as fraction of total cellular anti-TERT immunofluorescence signal) in MCF7 cells treated with the indicated concentrations of rapamycin. Bosutinib (1μM, red bars) was added to inhibit Src kinase. (*P<0.05, ANOVA for rapamycin concentration dependency and t-test for the effect of bosutinib at each rapamycin concentration) (**B**) Intracellular ROS levels (average DHR fluorescence intensity in flow cytometry, as percentage of untreated controls) in MCF-7 cells treated with rapamycin (concentrations indicated). B denotes additional treatment with 1 μM bosutinib. (* p<0.05, ANOVA, n=3-4 per condition) (**C**) Intracellular ROS levels in primary mouse ear fibroblasts from wild type (WT, left columns) and first generation TERT−/− (right columns) mice treated as in 5B. (2 way ANOVA, * p<0.05, for influence of rapamycin and genotype, n=7-8 per condition). Bosutinib treatment (middle column group) and lack of TERT (right columns) prevent decrease of ROS after rapamycin treatment in primary mouse ear fibroblasts, * p<0.05, One way ANOVA and Dunn's post hoc test, n=7-8 per condition.

To examine the effects of rapamycin on TERT shuttling from nucleus to mitochondria *in vitro*, we first treated TERT-positive MCF-7 human breast cancer cells with rapamycin for 3 days. We found a dose-dependent exclusion of TERT protein from the nucleus by rapamycin (Fig. 5A). Since TERT nuclear exclusion had been shown previously to be dependent on Src kinase [41] we applied the Src inhibitor bosutinib (1μM) to block TERT shuttling from the nucleus.

Indeed, rapamycin-driven shuttling of TERT was entirely dependent on the presence of functional Src kinase (Fig. 5A, red bars). Shuttling of TERT from nucleus to mitochondria reduced mitochondrial ROS production in multiple cell types [1, 2, 42]. Accordingly, a concentration-dependent decrease of intracellular ROS levels was found in rapamycin-treated MCF-7 cells which became significant at 200nM rapamycin (Fig. 5B). The reduction of ROS by rapamycin was completely diminished by the Src kinase inhibitor bosutinib (Fig. 5B).

In order to test the dependency of a ROS decrease on the presence of TERT as shown in brain mitochondria after rapamycin treatment *in vivo* (Fig. 4B, C) we employed mouse ear fibroblasts obtained from either wild type mice or first generation TERT knock-out mice and treated these cells with rapamycin for 3 days (Fig. 5C). We found the same rapamycin concentration-dependent decrease of ROS in TERT-positive wild type fibroblasts as in MCF7 cells. Moreover, inhibition of TERT shuttling by bosutinib again blocked the reduction of ROS by rapamycin. Importantly, rapamycin did not cause a decrease of ROS in fibroblasts lacking the TERT protein. Together, these results suggest that the beneficial effect of rapamycin on the decrease of ROS in TERT-positive cells depends at least partially on the capability of rapamycin to induce nuclear export of TERT protein into mitochondria, where TERT reduces ROS generation and/or release [1–3].

## DISCUSSION

Mitochondrial dysfunction and increased oxidative stress are associated with cellular senescence [17, 18] as well as aging [14]. We and others had previously shown that the telomerase protein TERT is able to improve mitochondrial function and to decrease oxidative stress [1–3]. In addition, it was demonstrated that although telomerase activity is downregulated early during human brain development [5, 7] and soon after postnatal development in mouse brains [6], TERT protein persists in adult mammalian brain and can enter neuronal mitochondria *in vivo* [10]. So far, oxidative stress, radiation or treatment with chemotherapeutic drugs such as etoposide have been shown to function as triggers for mitochondrial localisation of TERT in cellular models [1–3]. Recently Eitan et al. [9] showed that also glutamate stress *in vivo* can increase mito-chondrial TERT in Purkinje neurons.

It is well established that suppression of mTOR signaling can postpone aging and increase lifespan in multiple organisms [35, 39]. It is also known that mTOR is expressed in the developing and adult brain where it is involved various functions, such as synaptic plasticity, adult neurogenesis, memory, and learning [43- 45]. Haloran and colleagues showed that rapa-mycin treatment in mice improved brain function such as learning and memory in a Morris water maze [45]. The same group had already shown before that rapamycin treatment had beneficial effects on brain function and prevented cognitive decline of a mouse model of Alzheimer's disease [46]. We and others have demonstrated that rapamycin treatment can delay cellular senescence by improving mitochondrial properties by suppressing mitochondrial biogenesis and increasing retrograde signalling from mitochondria [18, 47].

Here, we show for the first time that DR and reduced mTOR signalling are physiologically relevant novel stimulators for mitochondrial TERT accumulation in brain *in vivo*, and TERT is causally involved in the effects of rapamycin on reduced mitochondrial ROS release. This result is in agreement with previous data of an inverse correlation between mitochondrial TERT localisation and mitochondrial ROS levels in different cell types *in vitro* including cultivated neurons [1–3, 10]. Cognitive function declines during aging [48]. This results in changes of learning and memory, which have been linked to the hippocampus [49, 50]. In agreement with these findings we demonstrate in this study that long term DR for 30 months improves spatial learning and short term spatial memory to a level indistinguishable from the younger mice while old AL mice at the same age as the DR mice were impaired in their learning ability. Although correlative, our results suggest that the changes in brain function might be associated to mitochondrial function and ROS release from the organelles.

Using a TERT−/− mouse model, we provide evidence that rapamycin mediated reduction in mitochondrial ROS release in brain tissue depends on the presence of TERT since we did not see a decreased ROS release in TERT−/− mice. First generation TERT knockout mice used here have similarly long telomeres as wild type mice [51], excluding telomere length-dependent effects as a cause of these differences.

Several factors can influence changes in mitochondrial function during aging and DR, and reduced mitochondrial ROS release by DR in brain may thus be at least partly explained by its effects on reduced mTOR signalling which promotes TERT protein to localise to mitochondria. There are a number of well-documented mechanisms by which the mTOR pathway is involved in mitochondrial homeostasis, such as regulation of mitophagy and protein synthesis [52]. Our data suggest an additional mechanism by which reduced mTOR signalling might improve mitochondrial quality and function, namely by directing TERT protein into the organelle. Several studies have shown that mito-chondrial TERT has various functions within the organelle [1–3, 42]. These include decrease of mito-chondrial superoxide and intracellular peroxides [1, 2], decreased apoptosis and less damage to mitochondrial DNA [1] and nuclear DNA [3], improved respiration and binding to mitochondrial genes [2, 42] and functioning as a reverse transcriptase within mito-chondria by using various mitochondrial RNAs as templates [42]. Thus, the telomerase protein TERT appears to have multiple functions within mitochondria that might improve mitochondrial function and decrease ROS release under DR or conditions of reduced mTOR signalling.

Furthermore, the effect of DR on changes in mitochondrial TERT abundance was absent in liver, suggesting there may be important roles of TERT specifically in brain as shown by Iannilli and colleagues through the existence of cytoplasmic TERT in a complex with RNA granules binding p15INK4B RNA and regulating it's translation in mature neurons providing another pro-survival function in these cells [53].

Modelling the decrease in ROS following rapamycin treatment *in vitro* we found that presence of TERT and its nuclear exclusion are both required for the reduction of ROS production by rapamycin. Using either TERT knock-out cells or blocking TERT nuclear exclusion with the Src inhibitor bosutinib abrogated the decrease of intracellular peroxides after rapamycin treatment. Together, our data suggest that the mTOR pathway might have a pleiotropic effect on the subcellular shuttling of TERT to mitochondria. The nuclear exclusion of TERT in our cellular model was quantitatively less pronounced than that caused by chronic, high oxygen partial pressure or acute hydrogen peroxide treatment [1, 3]. This might be due to a slow kinetics of TERT shuttling driven by mTOR suppression similar to the one observed under hyperoxia [1].

In addition, we demonstrate here that TERT levels decrease in brain tissue with age in mice at both mRNA and protein levels. In contrast, both DR and rapamycin feeding increased mitochondrial TERT but had no effects on TERT abundance in whole brain homogenates. It had been shown previously that various stimuli that mimic aspects of DR, such as resveratrol and exercise, can increase cellular telomerase activity and TERT expression in different human cell types such as endothelial progenitor cells *in vitro* and PBMCs *in vivo* [54, 55] and might thus have beneficial anti-aging effects. However, the DR mimetic rapamycin has been reported to decrease telomerase activity and TERT protein levels in cancer cells in the absence of transcriptional changes in TERT expression [56]. This could mean that the regulation of TERT expression after rapamycin treatment might be different in brain tissue *in vivo* where we found an increased TERT expression after rapamycin treatment in mice than in cancer cells *in vitro*. Several groups reported that TERT can form a complex with mTOR and other proteins such as PI3K/Akt, Hsp90 and S6 kinase in cancer cells [56, 57]. This complex might be involved in the upregulation of telomerase/TERT during transcription as well as post-transcriptionally during IL-2 activation of transformed immune cells [57, 58]. Interestingly, Sundin et al. [56] reported that rapamycin is able to disrupt this mTor/TERT/Hsp90/S6K complex post-translationally. However, it is not known whether similar complexes form also in other cell types and in tissues *in vivo* and if they do, whether they impact on the subcellular localisation of TERT.

Taken all our results together, we suggest that TERT protein might be a mediator of the beneficial effects of DR by improving mitochondrial function in brain through reduced mTOR signalling.

## MATERIALS AND METHODS

### Cells and treatments

MCF-7 cells (ATCC), were cultivated as described in [3]. Primary mouse ear fibroblast were isolated and cultivated as follows:

Mouse ear notches or whole ears were collected and stored in cold Serum Free Medium (SFM), Advanced DMEM/F12 (Life Technologies) chopped with a sterile scalpel and incubated in SFM with 1mg/mL of collagenase A (Roche) for 1.5h at 37°C in 5% O_2_. After digestion, the tissue was passed through an 18G syringe needle and then centrifuged for 10 minutes at 1000 rpm. Next, pellets were resuspended in advanced DMEM/F12 medium with 10% FBS (SIGMA), 2mM L-glutamine (GIBCO) and a mix of 50U/mL penicillin and 10mg/mL streptomycin (GIBCO). Isolated cells were incubated and cultivated at 37°C in 3% O_2_ and 5% CO_2_.

Cells were treated with 100 or 200nM rapamycin (SIGMA) in serum containing medium for 3 days. Controls were treated with the same amount of DMSO. Bosutinib (Santa Cruz Biotechnology) was also prepared in DMSO and controls treated with DMSO only.

### Mice and treatments

In the late onset dietary restriction study (experiment 1), the mouse strain ICRFa on a C57BL/6 background was used [59]. Mice used in DR experiments 2 - 4 were the inbred C57BL/6 strain from Harlan, (Blackthorn, UK). *mTert−/−* mice (strain:B6.129S-Tert, tm1Yjc/J, [60] were purchased form the Jackson Laboratory (USA) and were bred to obtain wild type, first generation (*F1*) knockout and heterozygote mice. Mice used in all experiments were group-housed in the same room and were provided with sawdust as well as paper bedding. Mice were accommodated at 20 ± 2°C under a 12 h light/12 h dark photoperiod with lights on at 7 am. All animals had *ad libitum* access to water. AL (*ad libitum*) fed mice had 24h access to standard rodent pelleted chow while the DR (dietary restricted) mice received a smaller pellet size (4 mm), both from Special Diets Services, Witham, UK. The reduction in food intake for the DR experiments was calculated from the food intake of the control AL group in each experiment [61]. Onset and duration of the experiments are listed in Table 1.

For rapamycin treatment 12 months old TERT−/− and wild type littermates received encapsulated rapamycin supplementation [38] for 4 months, thus mice were 16 months old when used. The dose of rapamycin was 14.7 mg per kg mouse as described in [38]. The experimental chow was a gift from Randy Strong (The University of Texas Health Science Centre) while controls received the same food without rapamycin encapsulation.

The project was approved by the Faculty of Medical Sciences Ethical Review Committee, Newcastle University. Experiments were conducted in accordance with UK Home Office legislation under the Animals (Scientific Procedures) Act 1986. The work was licenced by the UK Home Office (PPL 60/3864) and complied with the guiding principles for the care and use of laboratory animals.

### qPCR

Frozen mouse brains were ground with a customised tissue grinder in liquid nitrogen and stored at −80°C. RNA was isolated from 30mg of powder using RNeasy® Lipid Tissue Mini Kit (Qiagen) following the manufacturer's protocol. Reverse transcription was performed with 1μg of RNA and SuperScript® III Reverse Transcriptase (Invitrogen).

mTERT expression in the brain samples was analysed using Real-time Quantitative PCR and GAPDH was used as reference gene in the analysis using the SensiFAST™ SYBR® Hi-ROX (Bioline), on a Step One Plus instrument (Applied Biosystems) with the programme consisting of 95°C for 2 min, 50 cycles of 5 seconds at 95°C and 20 seconds at annealing temperature of each primer pair and a melt analysis step at gradually increased temperature from 68°C to 95°C. The expression of mTERT was normalised to the expres-sion of the housekeeping gene mGAPDH and the results were calculated and reported with the format of compara-tive threshold cycle (ΔΔCT). Sequence and annealing temperatures of primers used are detailed in Table 2.

**Table 2:** Primer sequences and PCR conditions for qPCR

Name	Sequence	Annealing temperature
mTERT forward	5′-GGATTGCCACTGGCTCCG	68°C
mTERT reverse	5′-TGCCTGACCTCCTCTTGTGAC	
mGAPDH forward	5′-GAACGGGAAGCTCACTGGC	62°C
mGAPDH reverse	5′-GACAACCTGGTCCTCAGTGT	

### TERT ELISA

TERT protein abundance in brain tissues and mitochondrial fractions was assessed using the TERT ELISA Assay (GenWay) including an internal standard. Cell pellets, homogenised or ground tissues and mitochondrial pellets were lysed in CHAPS buffer (Roche) and the ELISA performed with 100μg protein as described in [10]. Specificity was tested by using mouse ear fibroblasts from TERT−/− mice which did not show any signals.

### Generation of mouse brain homogenate and isolation of mitochondria

The method was performed as described [14]. In brief: Whole brains were placed in ice cold isolation medium (250 mM Sucrose, 10mM Tris, 0.5mM EDTA pH 7.4 at 4°C) immediately after killing the mice, and transferred to the Dounce glass tissue grinder to homogenise. An aliquot of the homogenates was immediately snap frozen and stored at −70°C. Brain mitochondria were isolated from the rest of the homogenates according to [62].

### Hydrogen peroxide release from mitochondria

The experiments were performed as published in [14, 63]. In brief, H_2_O_2_ release from isolated brain mitochondria was measured in assay buffer containing 115 mM KCl, 10 mM KH_2_PO_4_, 2 mM MgCl_2_, 3 mM Hepes, 1 mM EGTA, 0.2% fatty acid free BSA, pH 7.2 at 37°C in the presence of exogenous superoxide dismutase (75 U/ml), horseradish peroxidase (HRP) (2 U/ml) and Amplex Red (50μM, Thermo Fisher) at 37°C. The fluorescent intensity of resorufin, the oxidised product of Amplex Red, was monitored kinetically in a plate reader (FLUOstar Omega, BMG Labtech) at the excitation and emission wavelengths of 544 nm and 590 nm, respectively. Mitochondria were supplemented with either complex I (Pyruvate + malate, PM, 5mM) or complex II (succinate, 4mM). The maximum capacity of complex I to release H_2_O_2_ was determined in the presence of rotenone (5μM) with complex I linked substrate.

### ROS determination in cells using flow cytometry

Intracellular ROS levels of MCF7 and mouse ear fibroblasts were determined by staining of cells with 80 μM dihydrorhodamine 123 (DHR, Molecular Probes) and measuring in the green (FL1) and red (FL3) channels of a Flow cytometer (Partec, Muenster, Germany) as described in [1, 17].

### Immuno-fluorescence staining for hTERT in MCF7 cells

Immuno-fluorescence staining was performed using the TERT antibody (Rockland, USA) as described in [1, 3]. In brief: cells were grown on cover slips for 3 days and treated with 100 and 200nM rapamycin and DMSO for control. Cells were fixed with 4% PFA and stained with TERT primary and Alexafluor554 secondary antibody. At least 10 cells were analysed per condition and the amounts of TERT within the nucleus and cytoplasm determined using ImageJ software.

### Behaviour test: Barnes maze for spatial learning and memory

The Barnes Maze was used to assess spatial learning and memory which is predominantly determined by the hippocampus and is described in detail in [64]. Mice were allocated a specific target hole on a round disc with 20 holes and 4 visual clues on 4 sides. We did not use any adverse stimuli to promote mice to find their target holes (TH). On four consecutive days of learning 4 trials per day for 3 minutes each were performed. If mice did not find their allocated target hole within that time a 30 extra seconds were added to their time. For assessing and scoring the short and long term memory on days 5 and 12, respectively, mice were given one trial of 90 seconds to find their TH, which did not offer the escape box beneath the table. For failed attempts to find the TH an additional 20 seconds were added to the 90 seconds. In addition to the time to allocate the TH, a hole score was determined as the ratio between the sum of the value of each hole where mice poked their head into (10 for the target hole and one less for each consecutive neighbour-ing hole) and the number of total head pokes into holes.

### Statistical analysis

For the comparison of 2 groups Student's t-test was applied. To examine statistical significance of normally distributed data One Way ANOVA and Two Way ANOVA tests were performed with appropriate post-hoc tests using Sigmaplot^®^ software. Non-parametric data sets where tested by One Way ANOVA on ranks or Mann-Whitney Rank sum test. All data are presented as mean and error bars are standard error of the mean (S.E.M) unless otherwise stated. Results were considered statistically significant when the p value was <0.05.

## SUPPLEMENTARY MATERIALS FIGURES AND TABLES


