# Supplementary material for: Decreased mTOR signalling reduces mitochondrial ROS in brain via accumulation of the telomerase protein TERT within mitochondria
Source: Aging (Albany NY). 2016 Oct 22;8(10):2551–64. doi: 10.18632/aging.101089 (PMC5115906; doi:10.18632/aging.101089)
Supplement: Supplementary file 1 [file aging-08-2551-s001.pdf]

SUPPLEMENTARY MATERIAL

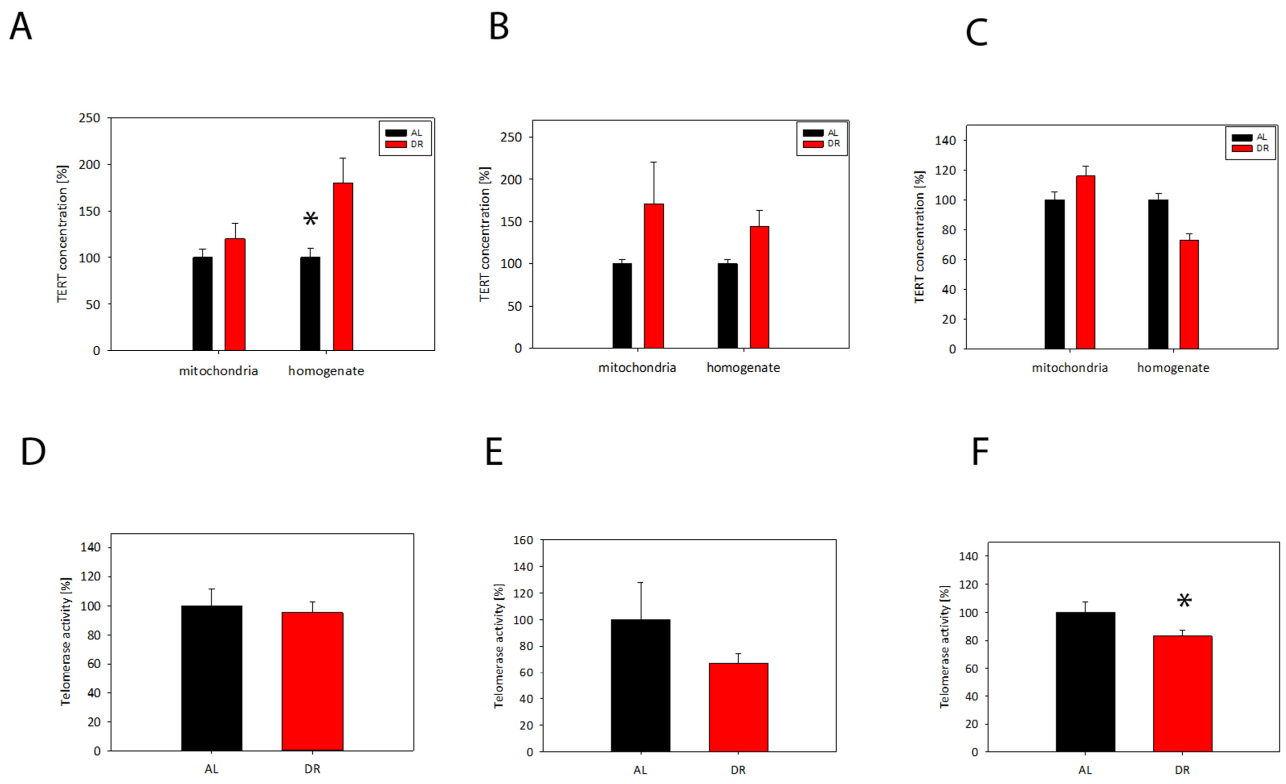

Supplementary Figure S1. TERT protein abundance and telomerase activity in mouse liver fractions of 3 short term DR experiments. (**A-C**) TERT protein abundance in liver mitochondria and homogenates from the same mice as used for figure 3 D-F (percentage of *ad libitum*-fed controls). (**D-F**) telomerase activity (TA, measured by TRAP assay as percentage of *ad-libitum*-fed controls) in liver homogenates in the same DR experiments as above. (**A, D**) Experiment 1; 4 animals per group. (**B, E**) Experiment 2, n=4 per group. (**C, F**) Experiment 3, n=7 per group for mitochondria and n=3 per group for homogenates. *Statistical significance was tested by t- test. * $p<0.05$.

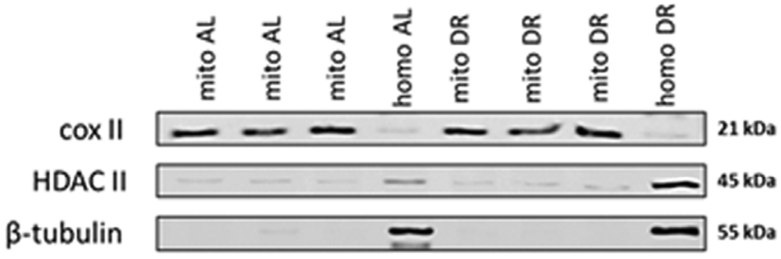

Supplementary Figure S2. Purity of the brain mitochondrial fraction. Isolated mitochondria and tissue homogenates were subjected to Western blotting with antibodies localized to mitochondria (Cox II, cytochrome c oxidase II), the nucleus (HDAC II, histone deacetylase II) and the cytoplasm (β-tubulin). Mito: mitochondrial fraction; homo: whole brain homogenate.

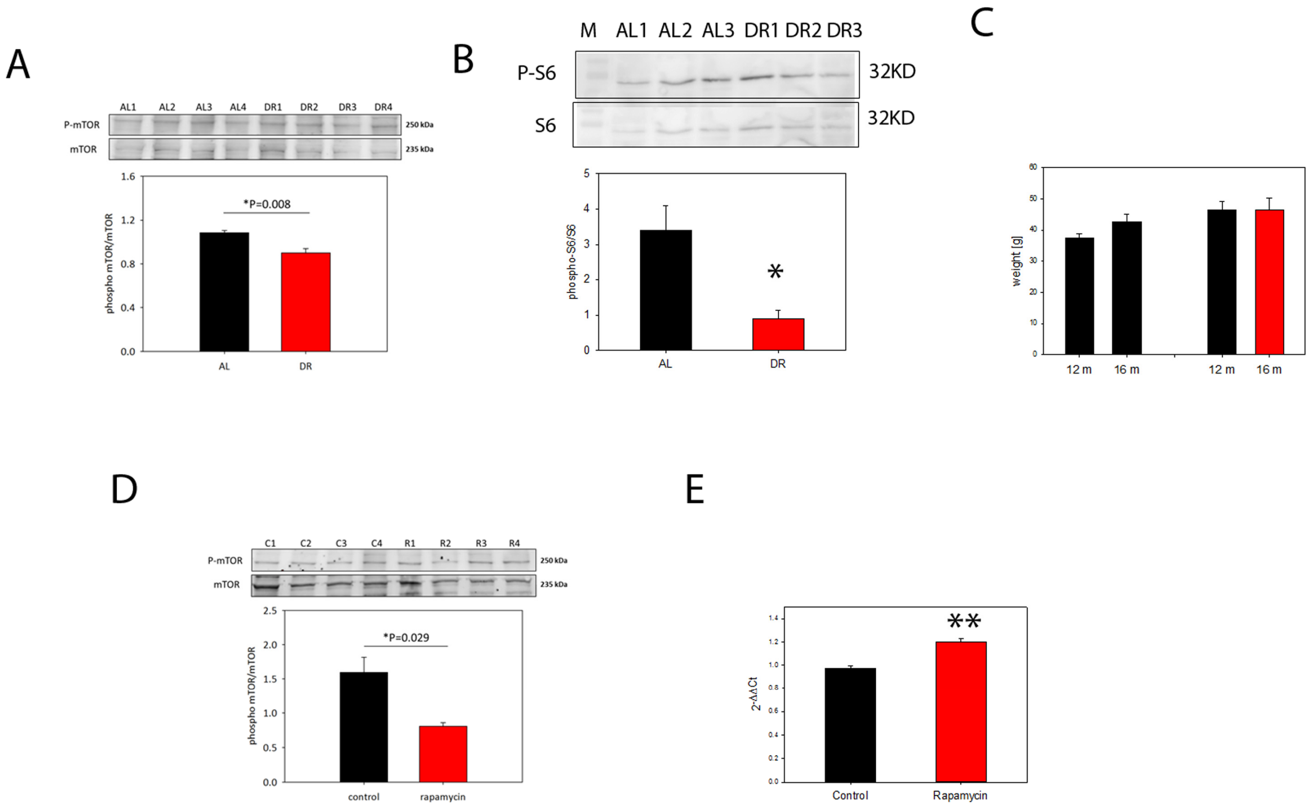

Supplementary Figure S3. Downregulation of the mTOR pathway in mouse brain after rapamycin treatment and short term DR. (**A**) mTOR phosphorylation after short term dietary restriction in mouse brain tissue after short term DR (exp. 1). Upper panels: immuno-blot with indicated antibodies, AL1-AL4 – *ad libitum* fed, DR1-DR4 – dietary restricted; lower panels: densitometric quantification, as the average of the ratios of phosphorylated to non-phosphorylated protein. * p=0.008, t-test. (**B**) S6 phosphorylation in the same experiment as in A. *P<0.05, t-test. (**C**) Body weight (in g) of rapamycin-treated and control wild type mice at ages of 12 months (before rapamycin) and 16 months (after rapamycin). (**D**) mTOR phosphorylation in brain tissue after 4 months of rapamycin treatment, C1-C4 –controls R1-R4 –rapamycin. * P<0.05, t-test (**E**) TERT mRNA abundance in mouse brain tissue after 4 months of rapamycin treatment measured by qPCR, n=3-4 per group. **P<0.001, t-test.

SUPPLEMENTARY METHODS

Immunoblotting

Tissue homogenates and mitochondria were lysed using CHAPS buffer (*Roche*). 60µg protein per sample was run on 10% polyacrylamide gel and blotted to ECL membrane (GE Healthcare) at 100V for 90 min. at 4°C, blocked and incubated with the primary antibody diluted over night at 4°C, After washing with TBST buffer and incubation with a peroxidase labelled secondary antibody membranes were developed with t a chemiluminescence detection kit (GE Healthcare) images were taken with a LAS 3000® camera (Fujifilm) and quantified by densitometry and normalised to the loading control (β-tubulin) using AIDA® software (Raytest).

TRAP assay

Telomerase was determined using the TeloTTAGGG telomere repeat assay (Roche) as described previously [7, 10] using 100ng liver tissue per reaction and a serial dilution of Hela cells (100, 10, 1, 0.1ng) as controls.

Supplementary Table 1. Primary antibodies used for immunoblotting

antibody	manufacturer	dilution
HDACII	Abcam	1:800
CoxII	Santa Cruz	1:200
phospho-mTOR (Ser2448)	Abcam	1:500
mTOR	Abcam	1:500
phospho-S6	Cell Signalling	1:1000
S6	Cell Signalling	1:1000
β-tubulin	Abcam	1:1000
